# Response Surface of Speed-Loading Path to Grain Refinement during Current-Heating Compression at SAE 5137H Steel

**DOI:** 10.3390/ma15103484

**Published:** 2022-05-12

**Authors:** Guo-Zheng Quan, Kun Yang, Yan-Ze Yu, Xue Sheng, Zhi-Hang Wen, Chao-Long Lu

**Affiliations:** 1 Chongqing Key Laboratory of Advanced Mold Intelligent Manufacturing, School of Material Science and Engineering, Chongqing University, Chongqing 400044, China; yk9999026@sina.com (K.Y.); yyz6201314@sina.com (Y.-Z.Y.); shengxue0305@sina.com (X.S.); wenzhihang97@163.com (Z.-H.W.); 15853361650@163.com (C.-L.L.); 2 State Key Laboratory of Materials Processing and Die & Mould Technology, Huazhong University of Science and Technology, Wuhan 430074, China

**Keywords:** SAE 5137H steel, processing map, enhanced deformation mechanism map, speed-loading path

## Abstract

In thermal deformation of materials, grain refinement induced by dynamic recrystallization (DRX) is often pursued to obtain excellent mechanical properties. Here, the thermal deformation behaviors of SAE 5137H steel were investigated and characterized at temperature and strain rate range of 1123–1483 K and 0.01–10 s^−1^. Meanwhile, a design approach in speed-loading paths for grain refinement during current-heating compression was proposed, and these paths are linked to a typical three-dimensional (3D) response surface. Depending on the acquired stress–strain curves, the flow behaviors of this steel were analyzed and the typical 3D processing map was constructed to clarify the stable processing parameter domains during the continuous deformation process. Then, by the typical 3D processing map and microstructure observation, the 3D deformation mechanism map was constructed to connect the processing parameters and microstructural mechanisms. Subsequently, the 3D activation energy map was constructed to evaluate these deformation mechanisms, and the enhanced deformation mechanism map was constructed. Eventually, based on the enhanced deformation mechanism map, the speed-loading paths for SAE 5137H steel during current-heating compression were designed and they are mapped in a 3D response surface.

## 1. Introduction

SAE 5137H steel is extensively applied in automobiles, railways and ships due to its strong toughness and high strength [1]. For SAE 5137H steel, hot working processes such as forging, rolling et al. play a crucial role in its industries application. In such hot working processes of steels, the processing parameters including temperature, strain rate, and strain have a significant effect on the microstructures resulting in component mechanical properties [2,3]. By designing the processing parameter loading path from a chaotic system, the microstructural characteristics of steels can be adjusted to improve their mechanical properties. To obtain excellent mechanical properties, the uniform and refined microstructures induced by dynamic recrystallization (DRX) are often pursued in hot deformation [3]. However, it is a crucial issue to acquire the anticipated microstructures and even excellent mechanical properties through the processing parameter loading path design [2]. The theory of processing map proposed by Prasad et al. [4] according to a Dynamic Material Model (DMM) provides an effective solution. It can clarify the stable and unstable processing regions, and can also identify the processing parameter window grain refinement [5,6,7]. However, the design of optimal parameter loading path should not only ensure stable material flowing and anticipated microstructures, but also ensure easy deformation. Thus, deformation activation energy (*Q*), which characterizes the degree of forming difficulty, is introduced into processing maps. Lower *Q* values represent easier deformation [8,9,10].

To date, the conventional processing map based on DMM applies three evaluation indexes including strain rate sensitive indicator (*m*), power dissipation efficiency (η), and instability parameter (ξ) to clarify the stable and unstable processing parameter windows under a certain strain. The conventional processing map was extensively used to study the thermal deformation behaviors and identify the optimal processing parameters for different materials, such as titanium alloys [11,12], aluminum alloys [13,14], magnesium alloys [15,16], nickel-based superalloys [17,18], steels [19,20], etc. However, the identified optimal processing parameters were not evaluated by lower deformation activation energy. Petr et al. [8] investigated the correlation among flow stress, η, and activation energy evolution of Cr-Mo low-alloyed steel, and they regarded the activation-energy processing maps as a tool for selection of processing parameters. Oleksandr et al. [21] and Peng et al. [22] respectively identified optimized hot processing parameters of nickel-based superalloy and homogenized Al-Zn-Mg-Cu alloy by a constructed conventional processing map and activation energy map of them under different true strains. Additionally, Quan et al. [10] divided the processing parameter windows with DRX mechanism in an enhanced processing map, and clarified the desired processing parameter regions corresponding with DRX and lower activation energy. In a word, scholars have clarified the processing parameter windows with desired microstructural mechanisms at discrete strains, and some of them have evaluated the parameter windows by lower activation energy [9,19,20,21,22,23,24,25]. However, for continuous hot deformation, these identified windows are discrete and local since the processing maps and activation energy maps are constructed under discrete strains. Actually, for the thermal deformation process, it is dynamic and continuous; meanwhile, the evolution of the deformation mechanisms and activation energy is also dynamic. This means that the identification of processing parameters should consider the dynamic evolution of microstructural mechanisms and the continuous variation of activation energy.

It is desirable that the processing parameters are always located in the stable regions that correspond to DRX mechanisms and lower activation energy. In a hot working process, it is feasible to obtain desired processing parameters by designing the speed-loading path at a constant temperature. In this study, the hot deformation behaviors of SAE 5137H steel were investigated, and a design approach in speed-loading paths for grain refinement during current-heating compression was proposed. Firstly, the flow stress–strain behavior of this steel was analyzed and the 3D processing map including three evaluation indicators mentioned previously was constructed to clarify the stable and unstable regions during a continuous deformation process. Then, by relating the constructed 3D processing map and microstructure analysis, dynamic recovery (DRV) and DRX mechanisms were identified; moreover, their dynamic evolutions were illustrated in a 3D deformation mechanism map. Subsequently, the *Q* values at different processing parameters were calculated and their variations were mapped in the 3D activation energy map. Meanwhile, an innovative enhanced deformation mechanism map was constructed. Finally, the speed-loading paths were designed based on the established enhanced deformation mechanism map, and these paths were mapped on a 3D response surface.

## 2. Design Approach in Speed-Loading Path and Its Principles

### 2.1. Construction of an Enhanced Deformation Mechanism Map

In DMM theory [4], a thermo-plastic deformation process is viewed as an energy dissipation course. The total absorbed power (*P*) expressed as Equation (1) is divided into one part (*G*) resulting from plastic deformation and the other part (*J*) caused by the microstructure evolution such as DRX, DRV, and phase transformation [26]. The strain rate sensitive index (*m*) determines the ratio of *J* and *G*, and it can be calculated by Equation (2) [26]. The power dissipation efficiency (η) is associated with *m* as expressed in Equation (3) [4]. Different η-values result from different deformation mechanisms containing DRV, DRX, superplasticity, and cracking [6]. Generally, higher η-value corresponds to a DRX softening mechanism, while high temperature wedge cracking also can lead to a higher η-value. Therefore, the instability criterion is introduced to further separate the unstable processing parameter domains. As expressed in Equation (4), negative ξ-values denote unstable deformation mechanisms [4]:

(1) $$P=\sigma \dot{\varepsilon }=J+G=\int_{0}^{\sigma }\dot{\varepsilon }d\sigma +\int_{0}^{\dot{\varepsilon }}\sigma d\dot{\varepsilon }$$

(2) $$m={\left(\frac{\partial J}{\partial G}\right)}_{T,\varepsilon }={\left(\frac{\partial \log\sigma }{\partial \log\dot{\varepsilon }}\right)}_{T,\varepsilon }$$

(3) $$\eta =J/J_{max}=\frac{2m}{m+1}$$

(4) $$\xi \left(\dot{\varepsilon }\right)=\frac{\partial \log\left(\frac{m}{m+1}\right)}{\partial \log\dot{\varepsilon }}+m<0$$

Depending on the above description of three evaluation indicators (*m*, η, and ξ), the conventional processing map is constructed. To reveal the continuous variation of these indicators in a thermo-plastic deformation process, a 3D processing map is constructed. By analyzing the microstructures at different forming conditions, the predominant microstructural mechanisms containing DRX and DRV are distinguished. Then, the critical η-values represent these mechanisms are confirmed. According to these different critical η-values in the constructed processing map, a 3D response space is developed, and it is called the 3D deformation mechanism map. From this, the dynamic evolution of microstructural mechanisms along with processing parameters is revealed.

The developed 3D deformation mechanism map can provide a solution to clarify the DRX processing parameter domains with desired refinement microstructures; however, considering the forming difficulty degree in a thermo-plastic deformation process, the identified parameter domains need to be further evaluated. Here, deformation activation energy is introduced.

Generally, the Arrhenius-type equation expressed as Equation (5) is employed to describe the relationships between processing parameters and activation energy [27,28]. For a certain strain, the average values of material constants $n^{\prime }$, β and n at different temperatures can be calculated by Equations (6)–(8) [22,29]. Then, the activation energy value (*Q*) can be calculated from Equation (9):

(5) $$\dot{\varepsilon }=A\left[\sinh\left(\alpha \sigma \right)\right]\exp\left(-\frac{Q}{RT}\right)$$

(6) $$n^{\prime }={\left(\frac{\partial \ln\dot{\varepsilon }}{\partial \ln\sigma }\right)}_{T}$$

(7) $$\beta ={\left(\frac{\partial \ln\dot{\varepsilon }}{\partial \sigma }\right)}_{T}$$

(8) $$n={\left(\frac{\partial \ln\dot{\varepsilon }}{\partial \ln\left(\sinh\left(\alpha \sigma \right)\right)}\right)}_{T}$$

(9) $$Q=R\cdot n\cdot \frac{\partial \ln\left(\sinh\left(\alpha \sigma \right)\right)}{\partial \ln\left(1/T\right)}$$

where $\dot{\varepsilon }$ is strain rate (s^−1^); *T* is deformation temperature (K); σ is flow stress (MPa); *Q* is the deformation activation energy (kJ·mol^−1^); *R* is the universal gas constant (8.314 J·mol^−1^·K^−1^); *A*, α, β, n, and $n^{\prime }$ are material constants; $\alpha =\beta /n^{\prime }$.

The *Q* values at different processing parameters are obtained respectively through the descriptions above. In addition, the continuous dynamically variation of activation energy is illustrated in a 3D activation energy map, in which the domains with lower and relatively stable *Q* value correspond to the processing parameter domains with stable flow and easy deformation. To identify the optimal processing parameters corresponding to DRX domains with lower and relatively stable activation energy, an enhanced deformation mechanism map is developed by superimposing the 3D activation energy map over the 3D deformation mechanism map. From this, the optimal processing parameters domains can be identified.

### 2.2. Design Procedures and Response Surface of the Speed-Loading Path

**Figure 1:**
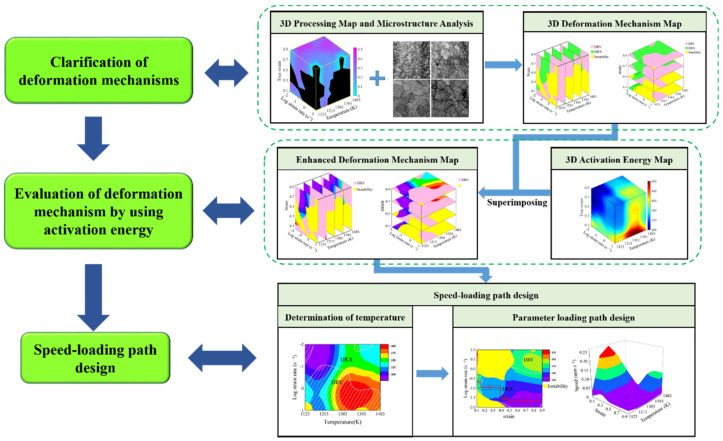
Procedures for the speed-loading path design.

Based on the above theory of the enhanced deformation mechanism map, procedures of speed-loading path design are proposed as Figure 1. Firstly, according to DMM theory, three evaluation indicators including strain rate sensitive index, power dissipation efficiency and instability parameter at different processing parameters are calculated. Meanwhile, the 3D processing map including these three evaluation indicators is established, in which the unstable and stable processing parameter domains are clarified. Secondly, by combining the η-value levels in the 3D processing map with microstructure analysis, the DRX and DRV mechanisms in stable domains are identified. Then, the 3D deformation mechanism map is developed to describe the correspondence between microstructure mechanisms and processing parameters. Thirdly, to further evaluate the identified parameter domains from the 3D deformation mechanism map, the activation energy values at these deformation parameters are calculated, and a 3D activation energy map is constructed. Fourthly, by superimposing the 3D activation energy map over the 3D deformation mechanism map, the enhanced deformation mechanism map is developed, in which the dynamic evolution of microstructural mechanisms and the continuous variation of activation energy can be revealed. Finally, based on the developed enhanced deformation mechanism map, the optimal processing parameters’ domains are identified, and then the optimal DRX parameter loading path can be designed. The detail design principles of parameter loading path are as follows: According to the criterion that optimal processing parameters correspond to DRX domains with lower and relatively stable activation energy, for different strains, the deformation temperature ranges are respectively separated from the enhanced deformation mechanism maps. The optimal temperatures are determined by selecting the same temperature values from these temperature ranges. For a specific optimal temperature, the continuous deformation process is divided into several stages according to strain extending and the variation of activation energy. Meanwhile, the strain rate ranges under these deformation stages are clarified from the enhanced deformation mechanism map. Then, based on the clarified strain rate ranges, the speed of compressing anvil can be calculated by Equation (10) [2], and the speed-loading paths are obtained by connecting the velocity at each stage. The scattered speed-loading paths linked to a 3D response surface, in which the optimal continuous parameters aiming to grain refinement and lower activation energy can be derived:

(10) $$v=h_{0}\dot{\varepsilon }\exp\left(-\dot{\varepsilon t}\right)$$

## 3. Materials and Experimental Procedures

**Figure 2:**
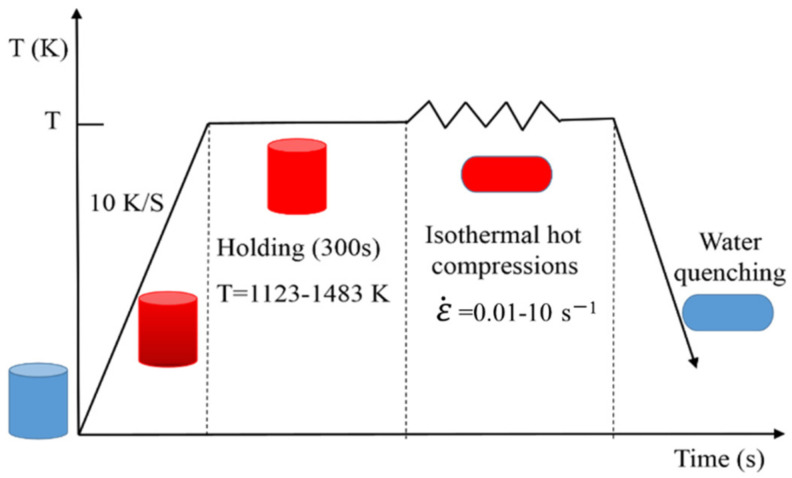
Schematic diagram of thermal compression tests.

The chemical composition of SAE 5137H steel in this study is listed in Table 1 by the vacuums emission spectrometer [1]. With the reference of ASTM standard: E209-00, twenty-six cylindrical with a diameter of 10 mm and a length of 12 mm were fabricated from one rolled billet. One of them was used as an original sample. To obtain basic computation data, twenty samples were compressed on a Gleeble-3500 simulator at temperatures of 1123 K, 1213 K, 1303 K, 1393 K, and 1483 K and strain rates of 0.01 s^−1^, 0.1 s^−1^, 1 s^−1^ and 10 s^−1^. The schematic diagram of compression tests was illustrated in Figure 2. From Figure 2, before compression, the samples were heated from an ambient temperature to the proposed deformation temperatures with a heating rate of 10 K/s, then maintained at that temperature for 5 min to obtain uniform temperature distribution and isotropous properties. Subsequently, the heated samples were compressed with constant temperature and strain rate until the height reduction ratio reached 60%. Finally, all of the deformed samples were quenched with water to ambient temperature rapidly to preserve the recrystallized microstructures. Considering the effect of strain, the second compression experiment was designed. Five samples were respectively compressed till height reductions of 10–50% corresponding to true strains of 0.105–0.693 at a temperature of 1303 K and strain rate of 0.1 s^−1^.

**Figure 3:**
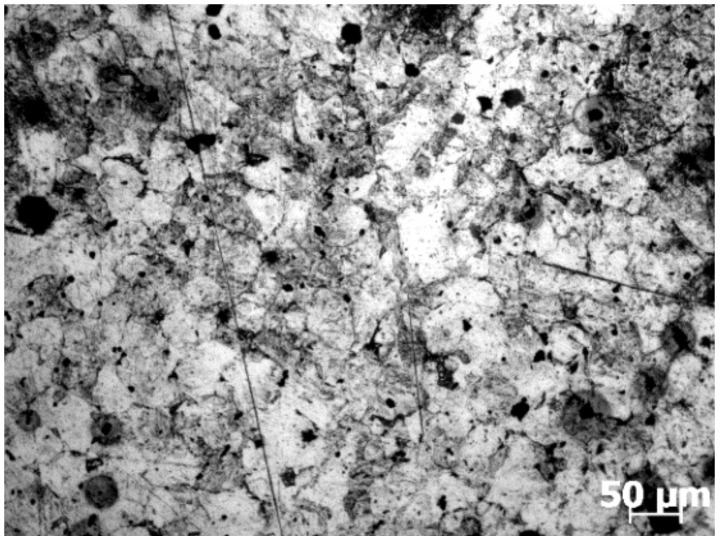
Initial optical micrograph of the rolled SAE 5137H steel sample.

After compression experiments, samples were sectioned perpendicular to the longitudinal compression axis for metallographic observation. Then, after grinding and polishing, all sections were etched in a 60 °C abluent solution of saturated picric acid. The treated sections were eventually observed by optical microscopy. The initial microstructure of the rolled SAE 5137H steel sample was shown in Figure 3. From the micrograph, the severely deformed microstructure after rolling can be observed.

## 4. Clarification and Evaluation of Deformation Mechanisms

### 4.1. Flow Stress–Strain Behavior

**Figure 4:**
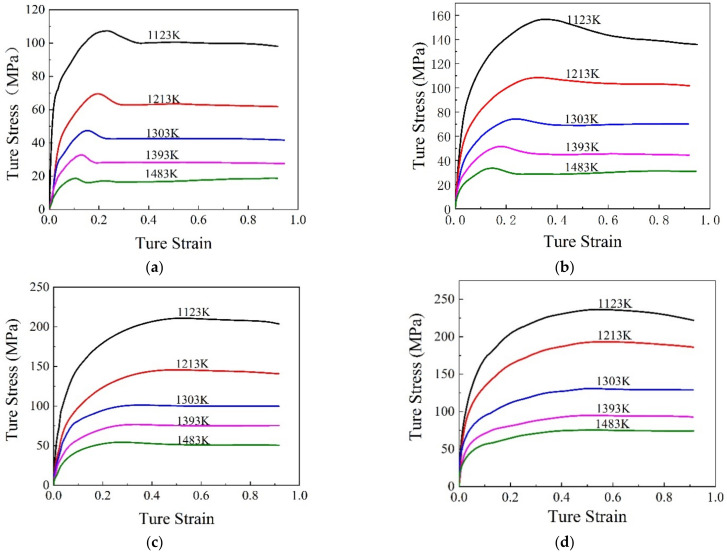
True stress–strain curves of SAE 5137H steel under different deformation temperatures with strain rates (**a**) 0.01 s^−1^, (**b**) 0.1 s^−1^, (**c**) 1 s^−1^, (**d**) 10 s^−1^.

The obtained stress–strain curves of SAE 5137H steel at different temperatures and strain rates were pictured in Figure 4. Generally, the trend of flow stress curves is mainly attributed to the dynamic competition relationship between work hardening (WH) and flow softening. From Figure 4, it is apparently found that the flow stress curves can be divided into the following two types, i.e., DRV-type and DRX-type. At temperature and strain rate range of 1123–1483 K and 1–10 s^−1^, the flow stress curves are considered as a DRV-type. During the initial stage of deformation, flow stress speedily increases to a statured value due to more WH and less DRV. Then, the saturated value sustains till the end of deformation indicating a balance between WH and DRV. However, at temperature and strain rate range of 1123–1483 K and 0.01–0.1 s^−1^, the flow stress curves are deemed as DRX-type. At the beginning of deformation, flow stress increases rapidly due to the effect of WH. With the rise of strain, DRX occurs at a critical strain, which leads to the softening effect strengthening, flow stress increases slowly and peak stress appears when the WH and dynamic softening reach an equilibrium. Subsequently, as strain rises further, the flow stress follows by a flow softening behaviors caused by DRX and DRV. Additionally, it is noted that the deformation temperatures and strain rates have a remarkable effect on the flow stress behaviors of SAE 5137H steel. The flow stress curves drop evidently with the increase of temperature and the decrease of strain rate. At higher temperature, the dislocations acquire enough energy for movement and annihilation, which ultimately decreases flow stress. At lower strain rate, the WH behavior becomes sluggish, thus exerting the decrease of flow stress.

### 4.2. 3D Thermal Processing Map

#### 4.2.1. Calculation of Three Indicators

**Figure 5:**
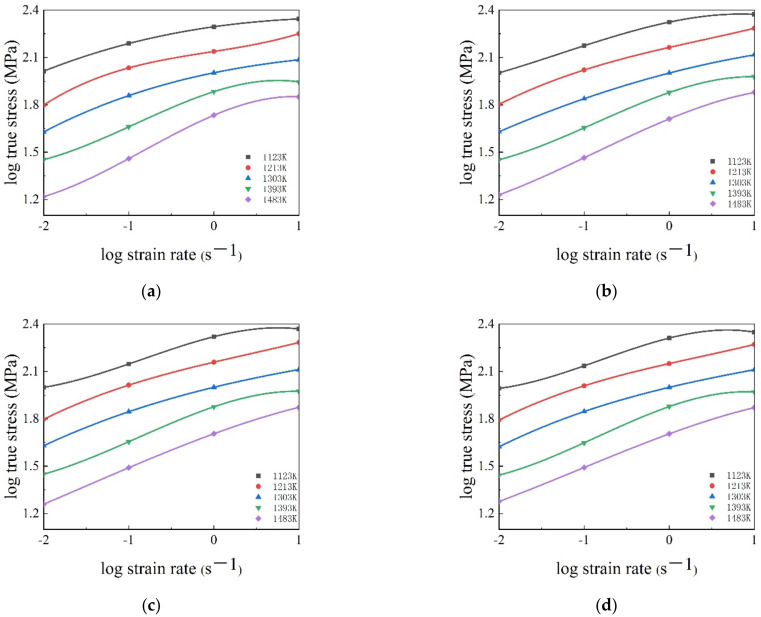
Relationships between stress and strain rate in log scale at different deforming temperatures and true strains of (**a**) 0.3, (**b**) 0.5, (**c**) 0.7, and (**d**) 0.9.

**Figure 6:**
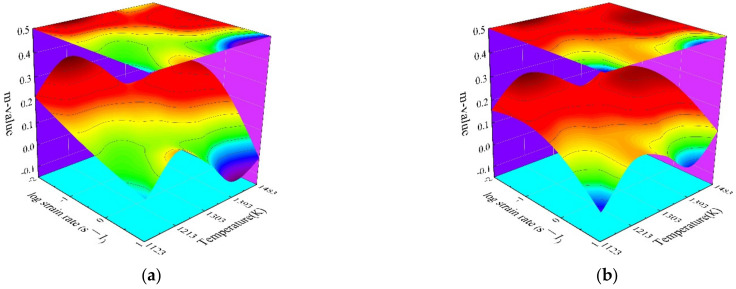

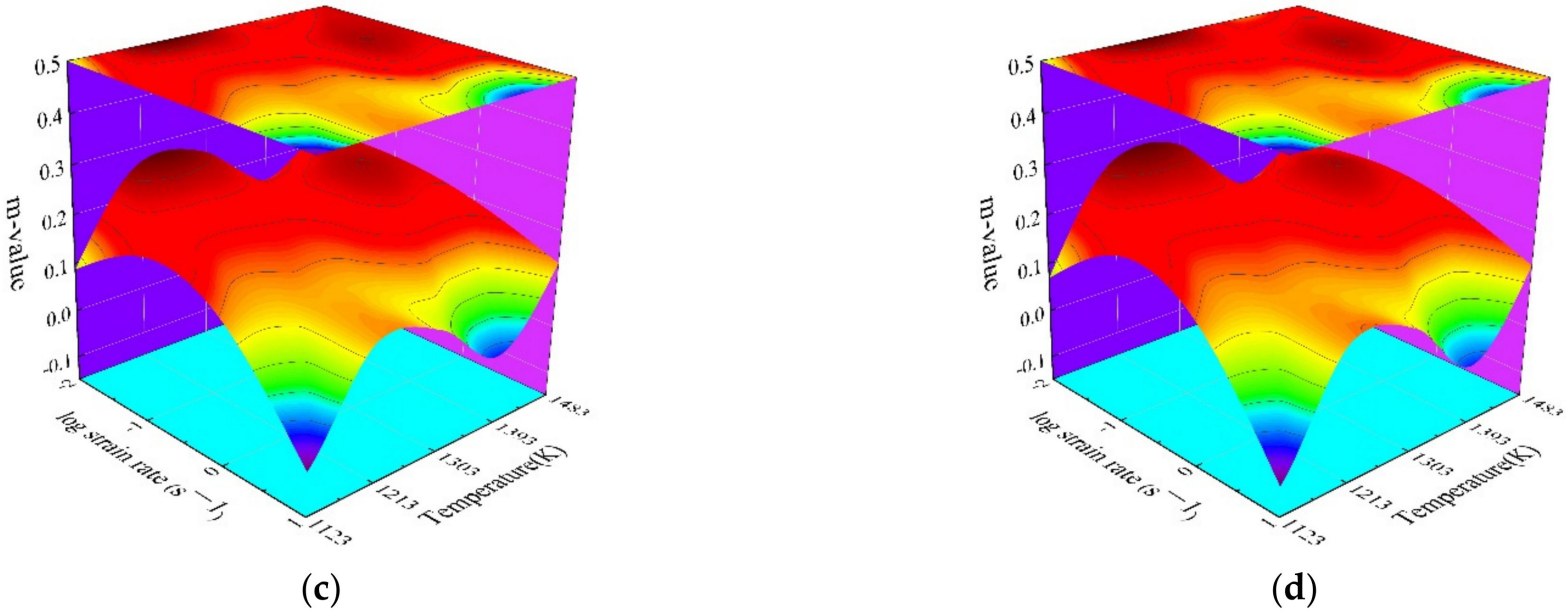
Strain-rate sensitivity index for SAE 5137H steel under different true strains of (**a**) 0.3, (**b**) 0.5, (**c**) 0.7, and (**d**) 0.9.

In the processing map, strain rate sensitive index, as a significant evaluation indicator, determines the power dissipation efficiency (*η*-value) and instability parameter (*ξ*-value) simultaneously. Based on the obtained stress–strain curves, the fitted cubic splines for $\dot{\log\sigma }$ versus $\dot{\log\dot{\varepsilon }}$ at different strains were illustrated in Figure 5. From Figure 5, the *m*-values at different forming conditions were calculated from the slopes of fitted cubic splines. Meanwhile, the smooth response surfaces between the forming conditions and m-values were illustrated in Figure 6. Generally, the variation of *m*-value corresponds to the transition of different deformation mechanisms. Some negative *m*-value can be observed under the following forming conditions: strain of 0.3, strain rate of 10 s^−1^, and temperature of 1393 K and 1483 K; strain of 0.5, 0.7 and 0.9, strain rate of 10 s^−1^, and temperature of 1123 K and 1393 K. As reported by Prasad et al. [6], the negative *m*-values usually represent the instability conditions. However, it is insufficient to accurately identify the stable processing parameter domains and clarify the specific microstructural mechanisms by only evaluating *m*-value. According to DMM theory, η and ξ were introduced to further identify the stable processing parameter domains and clarify deformation mechanisms.

**Figure 7:**
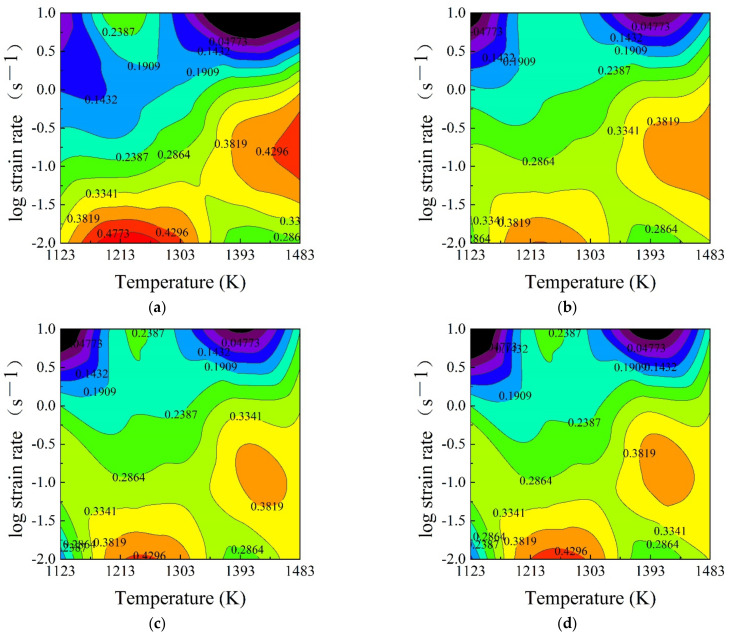
2D power dissipation maps for SAE 5137H steel under different true strains of (**a**) 0.3; (**b**) 0.5; (**c**) 0.7; and (**d**) 0.9.

**Figure 8:**
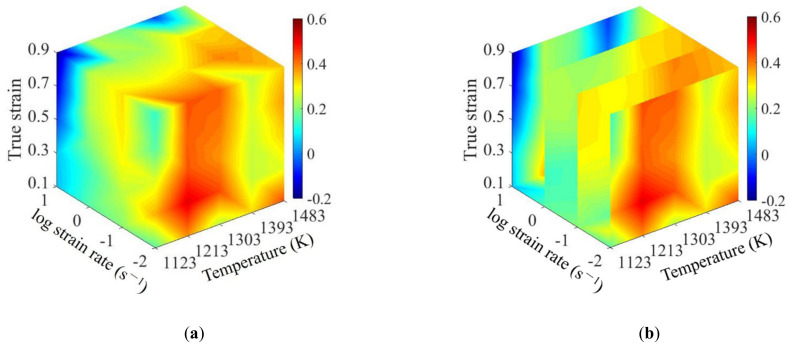
3D power dissipation maps: (**a**) three-dimensional body; (**b**) strain rate section.

The power dissipation efficiency (η-value) is considered as microstructural “trajectories”, which can characterize the dynamic microstructural evolution in a thermo-plastic deformation process. Based on the calculated *m*-values, the η-values were calculated using Equation (3), and the typical two-dimensional (2D) power dissipation maps at temperature-strain rate planes were constructed as shown in Figure 7. From Figure 7, the contour numbers indicate different η-values, and the regions with negative η-values indicate the unstable domains. However, at the discrete strains, the constructed 2D power dissipation maps are insufficient to reveal the continuous variation of η-value in the hot deformation process of SAE 5137H steel. Thus, by calculating the η-values at strains of 0.1, 0.2, 0.4, 0.6 and 0.8, taking temperature as the *x*-axis, log strain rate as the *y*-axis and strain as the *z*-axis, the 3D power dissipation maps were constructed as shown in Figure 8. It is observed from Figure 8 that the η-value remains at a higher level in the continuous parameter domain with temperature range of 1213–1483 K, strain rate range of 0.01–0.1 s^−1^ and strain range of 0.1–0.7. Conversely, η-value is lower or even negative in the continuous parameter domain with a temperature range of 1213–1303 K, strain rate range of 1–10 s^−1^, and strain range of 0.6–0.9.

As previously mentioned, according to the variation of η-value, the deformation mechanisms corresponding to different processing parameter can be clarified. Generally, the η-value corresponding to DRV mechanism is less than 0.25, the η-value related to DRX mechanism is about 0.3–0.45, and, when η-value exceeds 0.55, superplasticity may occur [4,17,18,19,20,21,22,23,24]. However, the processing parameter domains with a higher η-value are not always stable since an unstable deformation mechanism such as wedge cracking can also lead to higher η-value. Therefore, the instability parameter (ξ) was introduced to identify the unstable processing parameter domain.

**Figure 9:**
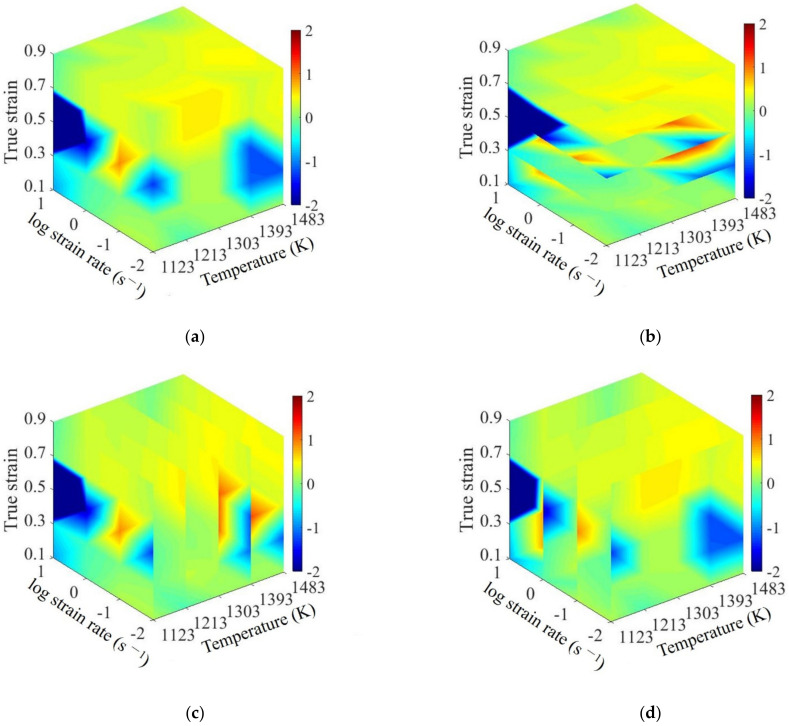
3D flow instability map: (**a**) three-dimensional body; (**b**) strain section; (**c**) temperature section; (**d**) strain rate section.

Based on all the calculated *m*-values, the instability parameters (ξ-values) at different forming conditions were calculated using Equation (4). In order to reveal the dynamic variation of ξ-value at different forming conditions, the 3D instability maps of SAE 5137H steel were constructed as shown in Figure 9. In Figure 9, the instability regions with negative ξ-value were identified, and the instability degree was characterized by graded colors. With the increase of stain, the instability regions decrease firstly then increase, and these regions mainly concentrate in low temperatures and high strain rates. This is attributed to the fact that the distortion energy of crystal lattice is relatively high under these conditions, which makes DRV and DRX mechanisms sluggish. To sum up, the variation of η-value and ξ-value is fundamentally attributed to the response of *m*-value to different forming conditions. Generally, the processing parameter domains with lower *m*-values are unstable. However, due to the complexity of the dynamic evolution of deformation mechanisms, the processing parameter domains with higher *m*-value are not always stable. Therefore, the stable and unstable domains need to be identified by comprehensively evaluating *m*-value, η-value, and ξ-value.

#### 4.2.2. Construction of the 3D Processing Map

**Figure 10:**
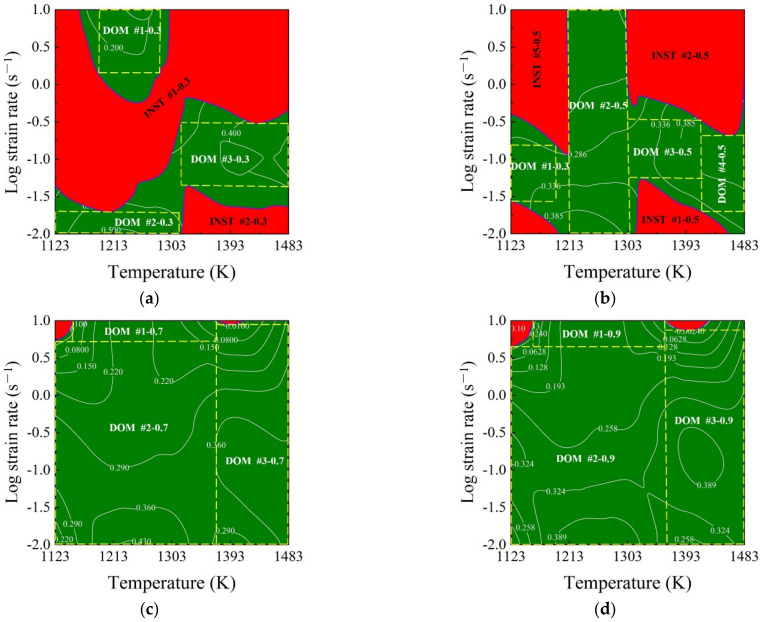
2D processing maps for different true strains of (**a**) 0.3; (**b**) 0.5; (**c**) 0.7; and (**d**) 0.9.

**Figure 11:**
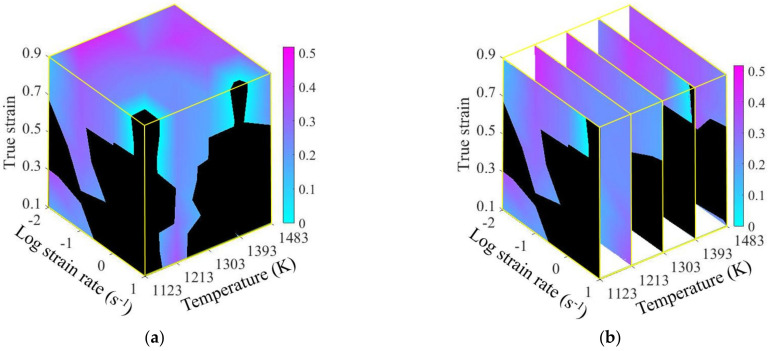
3D processing map: (**a**) three-dimensional body; (**b**) temperature section.

The conventional 2D processing maps of SAE 5137H steel at the strains of 0.3, 0.5, 0.7, and 0.9 were constructed by superimposing the instability maps over the power dissipation maps as shown in Figure 10. In each processing map, the contour numbers in the non-red areas represent η-value and the red areas correspond to unstable parameter domains. These unstable domains identified by comprehensively evaluating *m*-value, η-value, and ξ-value cover most of the microstructure defects in a thermo-plastic deformation process. Thus, these domains should be avoided when designing processing parameters. In Figure 10, the stable and unstable domains are divided by the rough blue curve, and different domains mark like “DOM” and “INST”, respectively. Taking the case at the strain of 0.3 as an example, the distribution of the stable domains was illustrated as follows: DOM #1—0.3 occurs in the strain rate and temperature range of 1.412–10 s^−1^ and 1195–1295 K; DOM #2—0.3 occurs in the strain rate and temperature range of 0.01–0.025 s^−1^ and 1123–1303 K; DOM #3—0.3 occurs in the strain rate and temperature range of 0.316–0.501 s^−1^ and 1303–1483 K. From the conventional 2D processing maps, the stable processing parameter regions at different discrete strains can be identified. However, for a continuous deformation process, it can not be guaranteed that the parameter loading path designed by 2D processing maps is always in the stable domains. Therefore, to reveal the continuous variation of stable domain in the continuous deformation process, a 3D processing map is constructed by superimposing the 3D instability map over 3D power dissipation map, as shown in Figure 11. In the 3D processing map, the black area represents unstable domains and the colored area represents stable domains; in addition, the color bar represents power η-value.

### 4.3. 3D Deformation Mechanism Map

**Figure 12:**
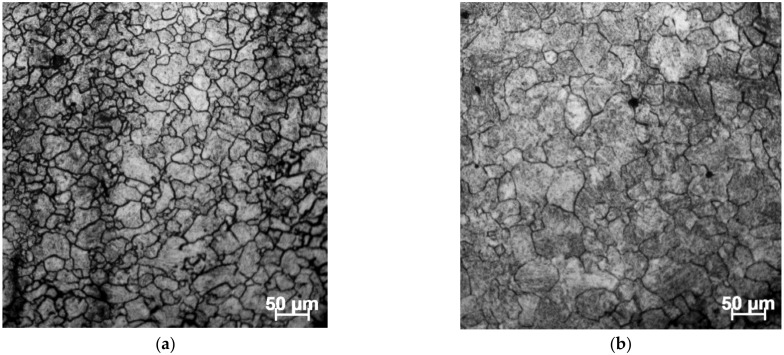

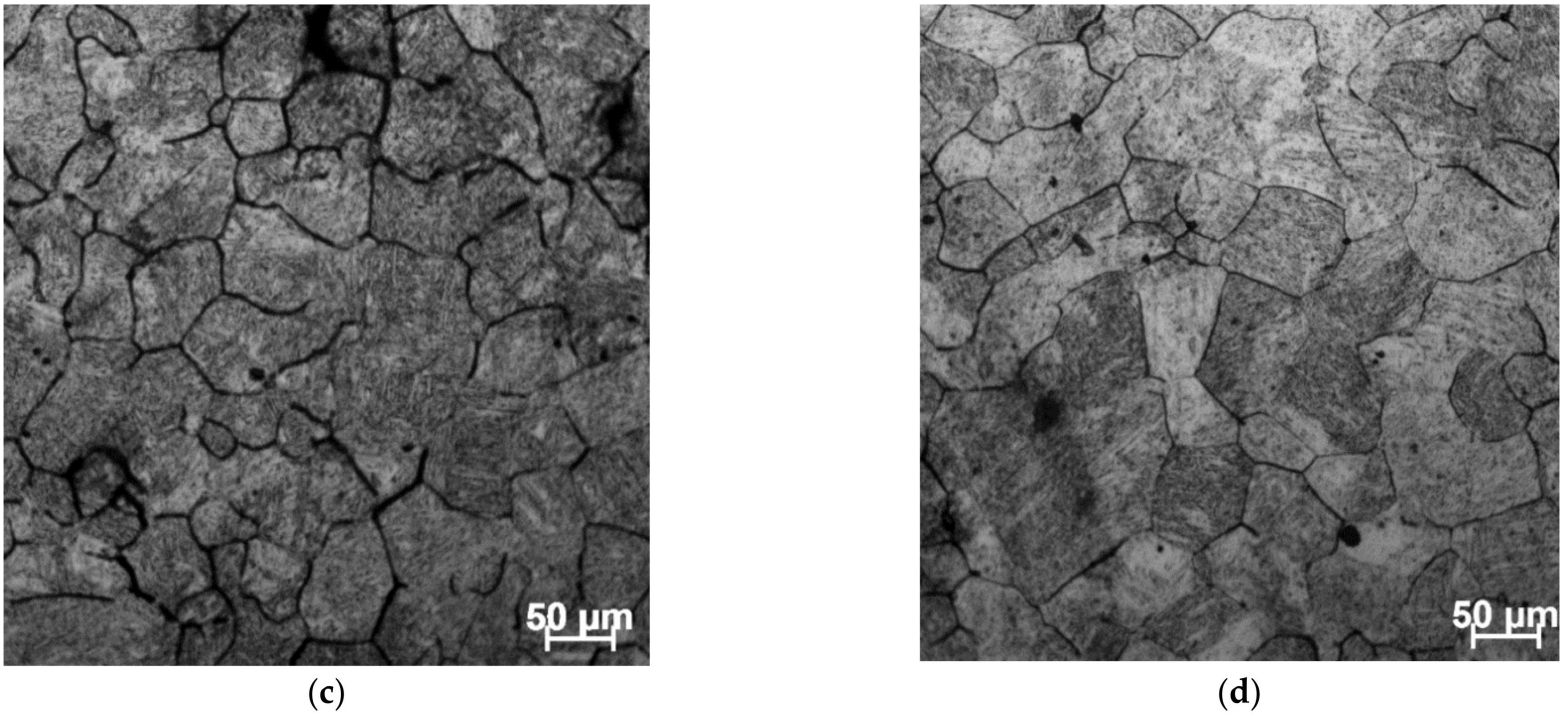
Optical microstructures of SAE 5137H steel in stable domains, a fix strain rates of 0.1 s^−1^ and different temperature: (**a**) 1213 K; (**b**) 1303 K; (**c**) 1393 K; and (**d**) 1483 K.

In order to validate the stable domains in the constructed 3D processing maps, meanwhile clarifying the internal relationship between microstructural mechanisms and processing parameters, the microstructures of deformed samples were characterized by an optical microscope. Figure 12 shows the microstructure of different processing parameters corresponding to the stable domain in 3D processing maps. Figure 12a presents the microstructure at the stable conditions of 1213 K and 0.1 s^−1^, corresponding to the η-value of about 0.324 in the processing maps. It can be observed from Figure 12a that some small grains are distributed around the large grains. This is a result of a mechanism in which, in deformation processes, the original grains elongate and the dislocation density increases; then, some sub-grains generated by dislocation polygonization are transformed into DRX grains distributed at the original grain boundary. Figure 12c,d represents the microstructure corresponding to the η-value range of about 0.32–0.38 in the processing maps. From Figure 12a–d, the grain size gradually increases with the rise of temperature when the strain rate is 0.1 s^−1^, which is attributed to the fact that DRX and grain growth play a dominant role at this time. Moreover, the grain size becomes larger with the rise of temperature. In Figure 12d, this coarse grain will not cause a fracture; however, it will deteriorate the metal properties and should be avoided during processing.

**Figure 13:**
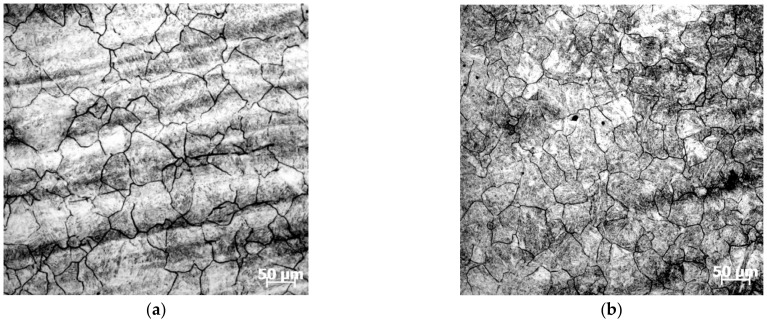

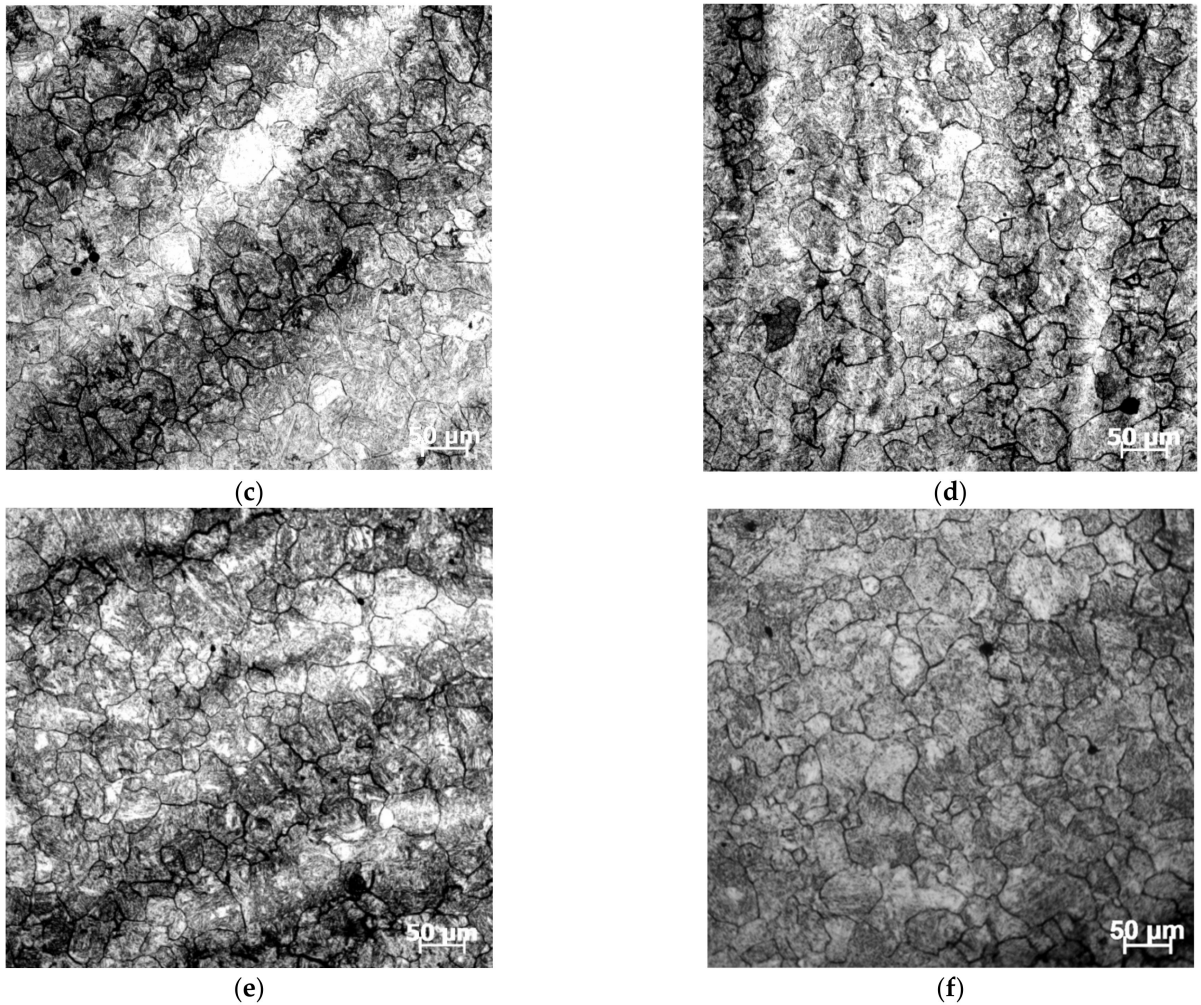
Optical microstructures of SAE 5137H steel at a constant temperature of 1303 K and strain rate of 0.1 s^−1^ under deformation degree:(**a**) 10%; (**b**) 20%; (**c**) 30%; (**d**) 40%; (**e**) 50%; (**f**) 60%.

The microstructures of SAE 5137H steel deformed to various deformation degrees of 10–60% at temperature of 1303 K and strain rate of 0.1 s^−1^ are shown in Figure 13a–f, respectively. As exhibited in Figure 13a, at the deformation degree of 10% (strain of 0.105), corresponding to the η-value of about 0.26, some equiaxed sub-grains were formed in the initial grains, which indicates the occurrence of DRV. When the deformation degree increased to 20% (strain of 0.223), as shown in Figure 13b, some small recrystallized grains formed and the grains size began to be uniform, which indicates the occurrence of DRX, and the η-value at this condition exceeds 0.3. With deformation degree further increasing to 50% (strain of 0.693), corresponding to the η-values range of 0.31–0.33, more and more recrystallized grains are observed in Figure 13c–e and the grains size tends to be more refined and uniform. As the strain increases, DRX has sufficient driven force to occur due to the increase of crystal defects and deformation stored energy. As the deformation degree increases to 60% (strain of 0.916), the effect of grain refinement gets closer to the maximum, and the distribution of grain size attains a relatively uniform level. It can be observed from Figure 13e,f that the degree of grain refinement has no remarkable difference at a deformation degree of 50–60%, which signifies that the samples have been approximately fully recrystallized at these conditions.

**Figure 14:**
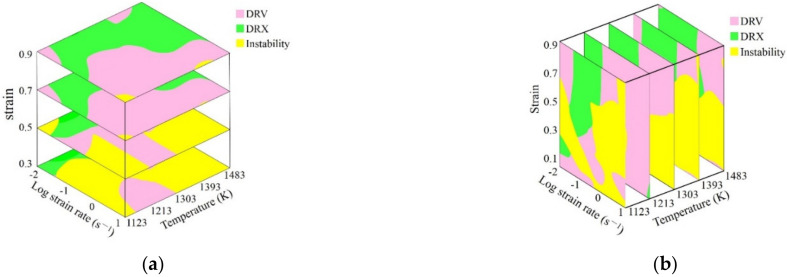
3D deformation mechanism map of SAE 5137H steel: (**a**) strain section; (**b**) temperature section.

Based on the above analysis, the conclusion was that it is available to identify the deformation mechanism by using the processing maps since the microstructure observation results correspond well with the constructed processing maps. Therefore, the 3D deformation mechanism maps of SAE 5137H steel, connecting processing parameter domains with deformation mechanisms, were constructed as Figure 14. In the 3D deformation mechanism maps, the yellow areas correspond to flow instability mechanism, and the green areas and pink areas correspond to DRV and DRX mechanisms, respectively.

### 4.4. Enhanced Deformation Mechanism Map

**Figure 15:**
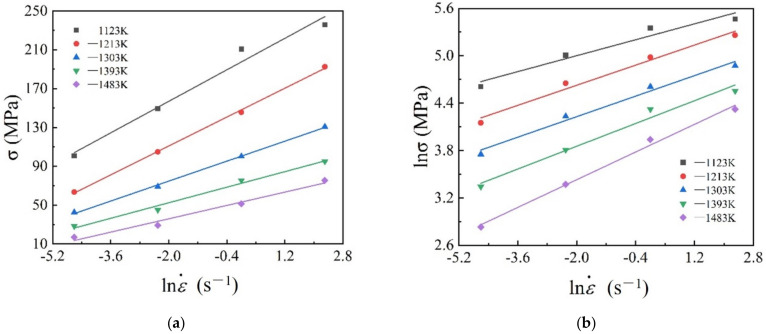

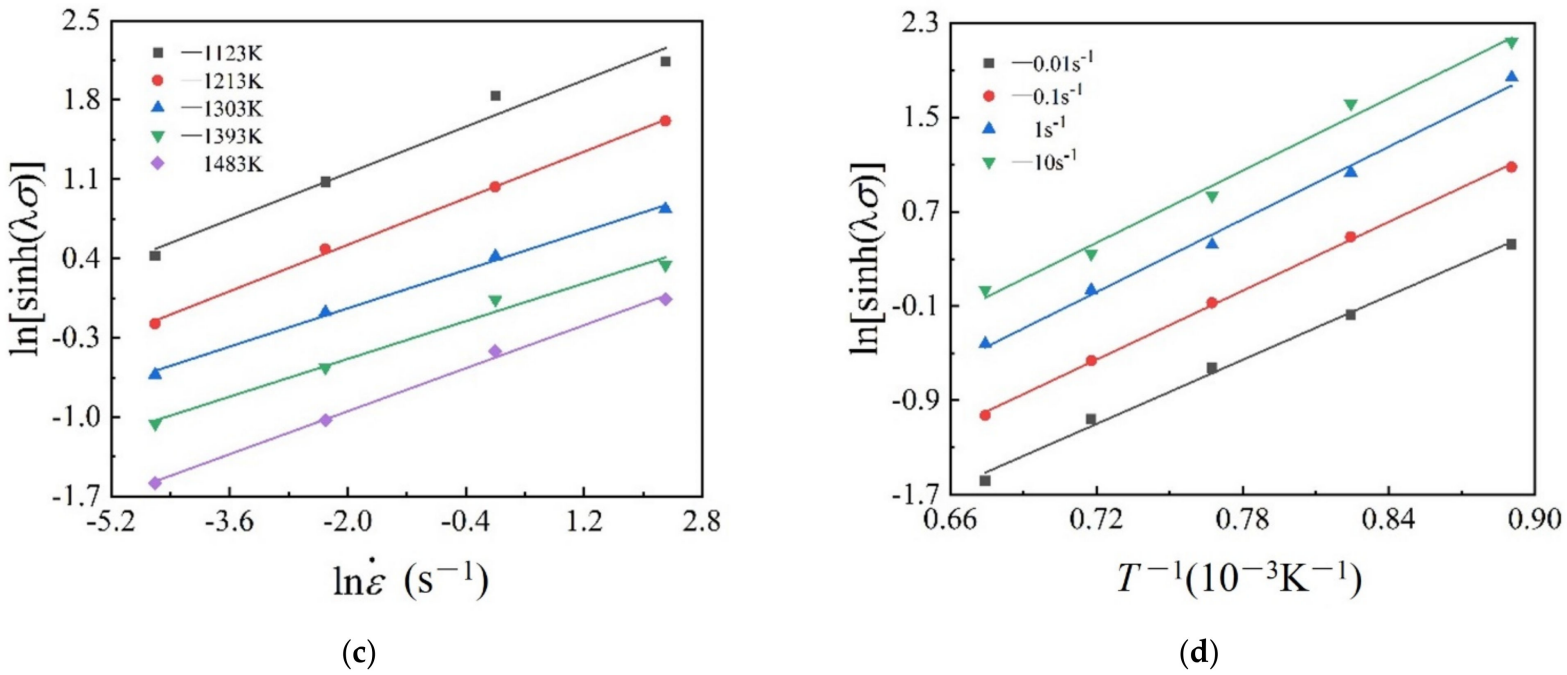
Linear relationships of (**a**) $\sigma -\ln\dot{\varepsilon }$; (**b**)$\;\ln\sigma -\ln\dot{\varepsilon }$; (**c**) $\ln\left[\sinh\left(\lambda \sigma \right)\right]-\ln\dot{\varepsilon }$; (**d**) $\ln\left[\sinh\left(\lambda \sigma \right)\right]-1/T$.

In this work, to evaluate the processing parameters during continuous deformation processes, *Q* values at different forming conditions were calculated, respectively. The following is taking the strain of 0.5 as an example to calculate *Q* values. At first, according to Equations (6) and (7), the linear regression of the relationships between lnσ, σ, and $\ln\dot{\varepsilon }$ (Figure 15a,b) at different temperatures were fitted out, and the mean slopes in two figures represent the reciprocal of $n^{\prime }$ and β, respectively. Here, β = 0.071099, $n^{\prime }\;$ = 5.908559, and α = 0.012033. Then, the linear regression of the relationships between $\ln\dot{\varepsilon }$, 1/T and $\ln\left(\sin h\left(\alpha \sigma \right)\right)$ could be plotted in Figure 10c,d. Finally, the deformation activation energy *Q* was calculated by Equation (9). Here, the average values of n and *Q* are 4.304415 and 364.52 kJ/mol, respectively.

**Figure 16:**
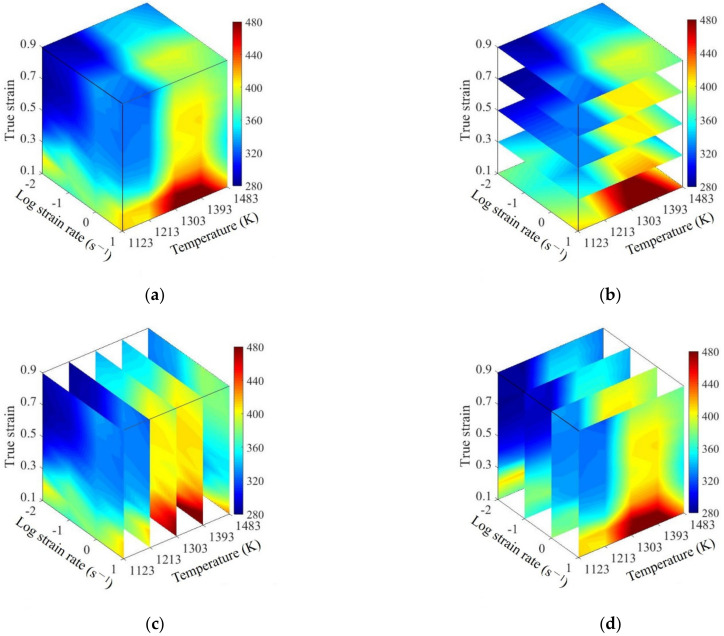
3D activation energy map of SAE 5137H steel: (**a**) three-dimensional body; (**b**) strain section; (**c**) temperature section; (**d**) strain rate section.

According to the calculated *Q* values at different forming conditions, the 3D deformation activation energy maps of SAE 5137H were established as shown in Figure 16. The evolution of *Q* at different strains is illustrated in Figure 16a,b. *Q* increases initially and then decreases rapidly. It results from the increase of energy barrier owing to work hardening at the initial stage; then, the energy barrier decreases resulting from the dynamic softening phenomenon as the strain continues to increase. It can be observed from Figure 16a,c that *Q* increases firstly and then decreases slightly with the rise of temperature, because the DRX is easier to be induced at this time. As the temperature increases further, the level of DRX and DRV can be improved, which helps to reduce the dislocation density. Therefore, *Q* value decreases since the resistance to dislocation motion is reduced. Additionally, from Figure 16a,d, *Q* increases gradually with the rise of strain rate during the whole thermal deformation. This is because the dislocation tanglement, which increases the energy barrier, can be aggravated by the rise of strain rates.

**Figure 17:**
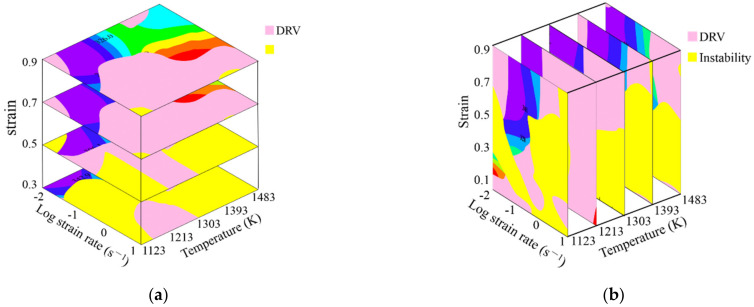
Enhanced deformation mechanism map of SAE 5137H steel: (**a**) strain section; (**b**) temperature section.

Based on the above work, an innovative enhanced deformation mechanism map, as illustrated in Figure 17, was constructed by superimposing a 3D activation energy map over a 3D deformation mechanism map. In Figure 17, the pink and yellow areas indicate DRV and instable regions, respectively, while the other areas are DRX regions and the numbers represent the values of activation energy. In DRX regions, the difficulty of its occurrence can be judged by the trend of *Q*, that is, the lower and more stable activation energy is, the more easily DRX occurs. Therefore, the optimum processing parameters can be determined by considering activation energy in the DRX region.

## 5. Design of Speed-Loading Path for SAE 5137H and Its Response Surface

**Figure 18:**
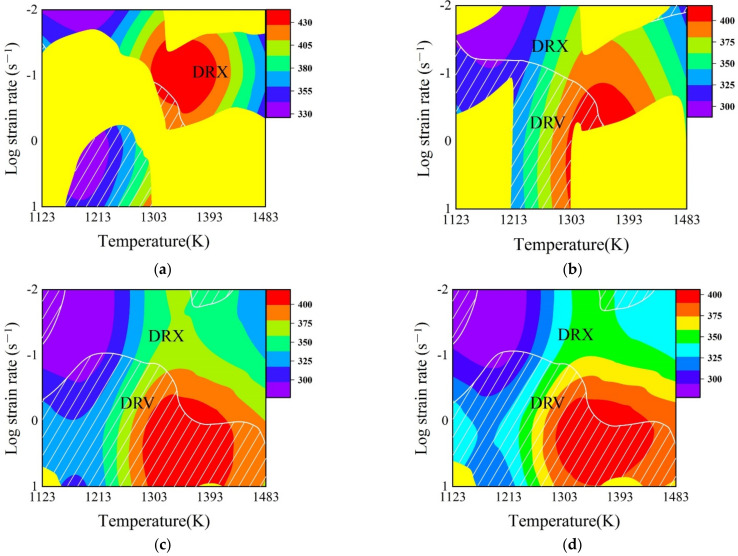
Enhanced deformation mechanism map of SAE 5137H steel under different true strains of (**a**) 0.3; (**b**) 0.5; (**c**) 0.7; and (**d**) 0.9.

**Figure 19:**
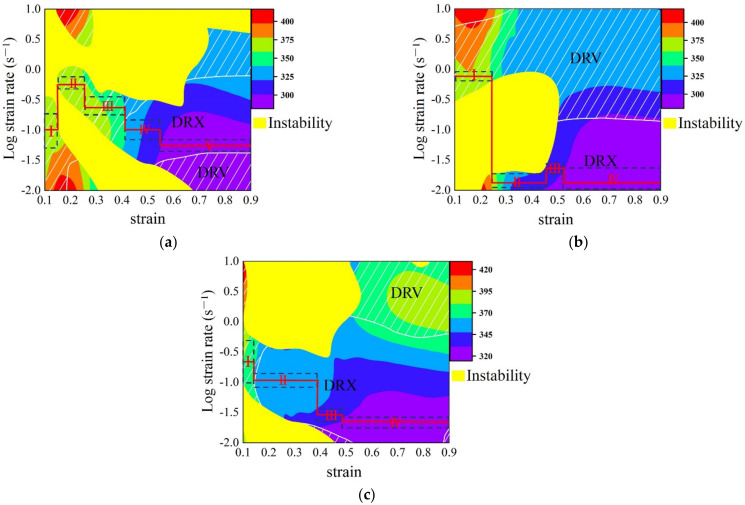
Enhanced deformation mechanism map of SAE 5137H steel at different temperatures of (**a**) 1123 K; (**b**) 1213 K; (**c**) 1483 K.

**Figure 20:**
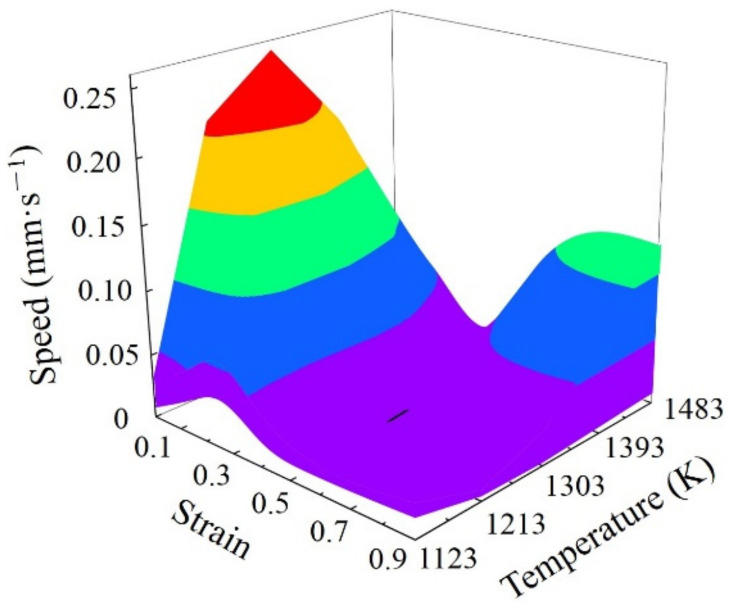
The 3D response surface of the speed-loading path.

The optimal DRX parameter loading path for SAE 5137H was designed based on the detailed procedures in Section 2.2. Figure 18 shows the enhanced deformation mechanism maps of SAE 5137H steel under four discrete strains. As displayed in Figure 18, the yellow areas and white line areas indicate instable and DRV regions respectively, and the other areas are DRX regions. Meanwhile, the color bar represents the *Q* values. Based on the principle of relatively low *Q*, the deformation temperature ranges under each strain were selected as shown in Table 2. Combined with the obtained temperature range under different strains, the continuous deformation temperatures were determined to be 1123 K, 1213 K, and 1483 K. The enhanced deformation mechanism maps at determined temperatures were proposed in Figure 19, and the deformation stages were clarified with a black dotted box. The division of deformation stages at different temperature was shown in Table 3. Taking the deformation temperature at 1123 K as an example, the method and basis for the division of deformation stages were illustrated in detail. It can be observed from Figure 19a that no DRX occurs when the strain is lower than 0.18. Moreover, the DRX region is very narrow and the *Q* in the region varies greatly in the strain range of 0.18–0.4. Therefore, the processing parameters should be in the DRV regions with low and stable *Q* when the strain is lower than 0.41. As shown in Figure 19a, the deformation process was divided into three stages as the strain is below 0.41. Stage I corresponds to the domain with the strain range of 0–0.15 and the strain rate range of 0.05–0.19. Stage II corresponds to the domain with the strain range of 0.15–0.25 and the strain rate range of 0.5–0.79. The average *Q* of Stage I and II is about 363 kJ/mol. Stage III corresponds to the domain with the strain range of 0.25–0.41 and the strain rate range of 0.17–0.4, with an average *Q* of about 358 kJ/mol. The rates of variation in *Q* between and within each stage remain extremely low. In addition, the DRX region is large when the strain is higher than 0.41, so the processing parameters should be in the DRX region with low and stable *Q*. The deformation process was divided into two stages according to the variation of *Q* as the strain is higher 0.41. Stage IV corresponds to the domain with the strain range of 0.41–0.55 and the strain rate range of 0.07–0.15, with an average *Q* of about 315 kJ/mol. Stage V corresponds to the domain with the strain range of 0.55–0.9 and the strain rate range of 0.05–0.07, with an average *Q* of about 298 kJ/mol. As shown in Figure 19b,c, the deformation processes at 1213 K and 1483 K were divided into several stages by using the same method. Subsequently, the compression speed at different strain stages was calculated by Equation (10), and the speed-loading path was obtained by connecting the velocity at each stage. The scattered speed-loading paths were linked to a 3D response surface as Figure 20, in which the optimal continuous parameters aiming for grain refinement and lower activation energy of SAE 5137H can be designed.

**Figure 21:**
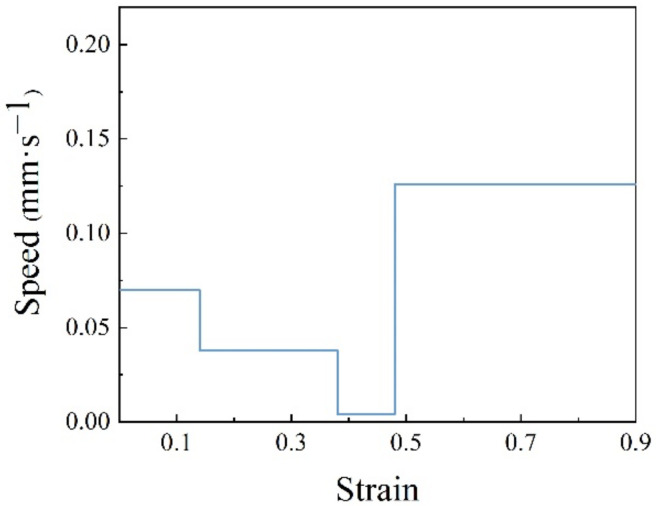
A speed-loading path at 1483 K.

**Figure 22:**
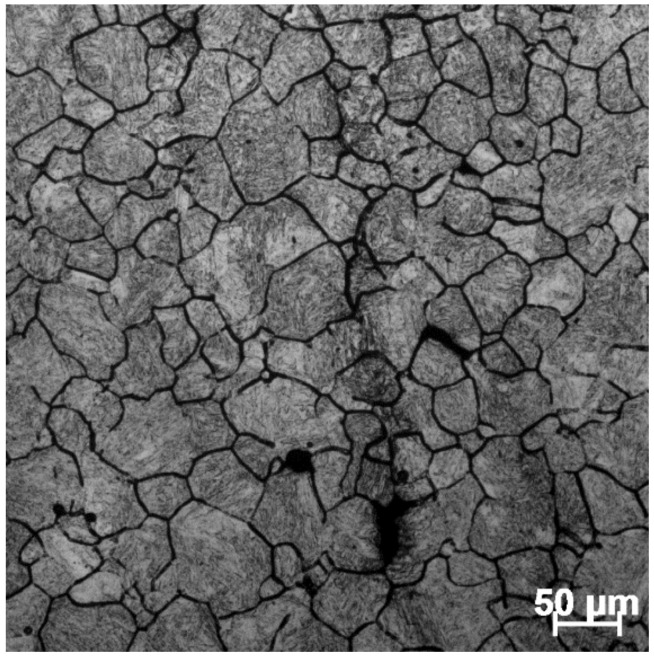
Optical microstructures of SAE 5137H steel corresponding to the speed-loading path at 1483 K.

In order to verify the effect of the 3D response surface, a speed-loading path at 1483 K was designed as shown in Figure 21. Based on the previous experimental steps, the microstructure at a corresponding compression condition was observed as shown in Figure 22. Compared with the microstructure at 1483 K and 0.1 s^−1^ shown in Figure 12d, it can be seen that the microstructure corresponding to the designed speed-loading path has a finer grain. It was concluded that the speed-loading path derived from the 3D response surface was beneficial and practical for grain refinement. Meanwhile, the processing parameters of the 3D response surface are always located in the lower activation energy domain.

## 6. Conclusions

The thermal deformation behaviors of SAE 5137H steel during current-heating compression were investigated and characterized. Then, speed-loading paths aiming for grain refinement and lower activation energy were designed by using the enhanced deformation mechanism map. The following conclusions of this work were obtained:

(1) Flow stress reduces with the increase of temperature and the decrease of strain rate. Additionally, flow stress shows obvious DRX characteristics at a strain rate of 0.01–0.1 s^−1^, while flow stress shows obvious DRV characteristics at a strain rate of 1–10 s^−1^.
(2) Based on the conventional 2D thermal processing map and considering the continuous deformation process, a 3D thermal processing map including a strain rate sensitive index, power dissipation efficiency, and instability parameter was constructed. By combining the established 3D thermal processing map with microstructural validation, the connection between deformation mechanisms and hot working parameters were illustrated in the 3D deformation mechanism map.
(3) The 3D deformation activation energy maps representing the difficulty degree of hot deformation were constructed. Activation energy value increases initially and then decreases with the rise of strain and temperature, while it increases gradually with the rise of strain rate. Meanwhile, an enhanced deformation mechanism map was constructed.
(4) The speed-loading paths of SAE 5137H were linked to a 3D response surface, which is applied to design the optimal continuous parameters aiming for grain refinement and lower activation energy. By experimental validation, the speed-loading path derived from the 3D response surface was beneficial and practical.

## Figures and Tables

**Table 1:** Chemical compositions of the studied SAE 5137H steel (wt%).

Element	Cr	Mn	C	Si	Ni	Mo	S	N	P
Content	1.23	1.19	0.38	0.28	0.11	0.042	0.025	0.015	0.015

**Table 2:** Range of deformation temperature under each strain.

Strain	0.3	0.5	0.7	0.9
Temperature (K)	1123–1289 & 1425–1483	1123–1303 & 1412–1483	1123–1313 & 1326–1483	1123–1303 & 1393–1483

**Table 3:** Division of deformation stages at 1123K, 1213K, and 1483 K.

Temperature (K)	Stage					
		I	II	III	IV	V
1123	Strain	0–0.15	0.15–0.25	0.25–0.41	0.41–0.55	0.55–0.9
	Strain rate (s^−1^)	0.05–0.2	0.479–0.75	0.174–0.37	0.066–0.15	0.04–0.067
1213	Strain	0–0.25	0.25–0.46	0.46–0.52	0.52–0.9	
	Strain rate (s^−1^)	0.63–0.85	0.012–0.02	0.02–0.026	0.01–0.023	
1483	Strain	0–0.14	0.14–0.38	0.38–0.48	0.48–0.9	
	Strain rate (s^−1^)	0.1–0.5	0.07–0.158	0.023–0.04	0.018–0.03	

## Data Availability

Not applicable.
